# Combining thiopurine with partial enteral nutrition promotes complete mucosal healing in pediatric Crohn's disease

**DOI:** 10.1111/ped.70290

**Published:** 2026-01-14

**Authors:** Emiri Kaji, Atsushi Yoden, Takahiro Namba, Satomi Nishimoto, Masano Otani, Takeru Okuhira, Keisuke Inoue, Tomoki Aomatsu, Daisuke Nishioka, Akira Ashida

**Affiliations:** ^1^ Department of Pediatrics Osaka Medical and Pharmaceutical University Takatsuki Osaka Japan; ^2^ Department of Pediatrics Kawanishi City Medical Center Kawanishi Hyogo Japan; ^3^ Department of Pediatrics Osaka Saiseikai Suita Hospital Suita Osaka Japan; ^4^ Okuhira Nobinobi Children's Clinic Mishima Osaka Japan; ^5^ Inoue Children's Clinic Hirakata Osaka Japan; ^6^ Aomatsu Pediatrics Clinic Takatsuki Osaka Japan; ^7^ Department of Medical Statistics, Research & Development Center Osaka Medical and Pharmaceutical University Osaka Japan

**Keywords:** maintenance therapy, partial enteral nutrition, pediatric Crohn's disease, thiopurine

## Abstract

**Background:**

The combination of thiopurines and partial enteral nutrition (TP/PEN) is a common maintenance therapy for mild‐to‐moderate Crohn's disease (CD) in pediatric patients. However, no studies have investigated the efficacy of TP/PEN. This study aimed to evaluate the non‐relapse and complete mucosal healing rates in pediatric CD patients following treatment with or without TP, in addition to PEN.

**Methods:**

This retrospective observational study included 39 pediatric patients diagnosed with mild‐to‐moderate CD. Based on the proposed treatment, the patients were divided into the TP/PEN and PEN‐only groups. The primary outcome was the relapse‐free rate between the two groups. Relapse was defined as a pediatric Crohn's disease activity index (PCDAI) >12.5, Simple Endoscopic Score‐CD (SES‐CD) score >7, Lewis score > 135, and/or abnormal C‐reactive protein levels (CRP > 0.15 mg/dL). The secondary outcome was complete mucosal healing (SES‐CD score 0) at 12–24 months.

**Results:**

Although the difference in non‐relapse rate between the two groups was not statistically significant, complete mucosal healing rates were significantly higher in the TP/PEN group than in the PEN‐only group. Complete mucosal healing at 12–24 months was achieved in 7/21 (33.3%) and 1/18 (5.5%) patients in the TP/PEN and PEN‐only groups, respectively (*p* < 0.05).

**Conclusion:**

Nowadays, more treatment options are available, and TP/PEN remains a viable option for the treatment of mild‐to‐moderate CD in pediatric patients.

## INTRODUCTION

Crohn's disease (CD) is a chronic inflammatory condition that may affect any part of the gastrointestinal tract from the mouth to the anus, characterized by periods of remission and relapse. CD developing during childhood severely disrupts the normal daily lives due to persistent abdominal symptoms, dietary restrictions, and frequent hospital visits. Compared with adult CD, pediatric CD often manifests as a more extensive and progressive condition, typically requiring more intensive treatment. Long‐term studies have shown that this condition may evolve into a more complex disease.^1^ Furthermore, pediatric patients with CD exhibit distinct features, including substantial growth retardation, delayed puberty, poor bone density, and weight loss, and some of these conditions are irreversible. The pathogenesis of these symptoms is complex, involving reduced dietary intake, increased gastrointestinal nutrient loss, and increased energy demands due to persistent inflammation. Uncontrolled inflammation leads to long‐term complications, including fibrotic strictures, enteric fistulae, and intestinal neoplasia. Therefore, the early and effective control of inflammation is crucial to mitigating adverse outcomes. The advent of new treatment methods, especially anti‐cytokine therapy such as anti‐TNF therapy, has considerably changed the management of CD.^2^ Although treatment options have become more diverse in recent years, the loss of response during anti‐TNF therapy remains a major issue. Despite its effectiveness in the treatment of more severe pediatric CD, a certain number of patients with CD who exhibited a good initial clinical response will eventually experience loss of response during therapy, occurring even within the first few months of treatment initiation.^3^ The loss of response to anti‐TNF therapy could partially be attributed to the low trough serum concentrations and the development of neutralizing antibodies during the maintenance phase. Although the treatment options have expanded, the number of available treatments is limited. Currently, infliximab is the most effective drug, and patients who develop resistance to infliximab are required to change to a slightly less effective drug. For children with CD, prolonged drug therapy is often necessary to maintain long‐term remission while maintaining treatment options. Restoring growth and promoting pubertal development while ensuring symptom‐free living are key treatment goals in pediatric CD. Recently, mucosal healing has been emphasized in addition to these goals. This has become a realistic therapeutic goal and is widely accepted, as it leads to fewer hospitalizations, surgeries, the need for systemic steroids, improved long‐term outcomes, reduced subsequent disease activity, and the risk of bowel damage. Complete mucosal healing (defined as simple endoscopic score‐CD [SES‐CD] of 0) is strongly associated with higher rates of long‐term clinical remission and continuous mucosal healing.^4^, ^5^, ^6^ Unlike ulcerative colitis, surgery is not a radical cure, and surgery for pediatric CD is recommended for removing lesions that cause symptoms such as fistulas and stenosis; however, mucosal inflammation often worsens at the surgical site. Therefore, surgery should be avoided whenever possible. Guidelines^7^ suggest that maintenance treatments for pediatric CD without a high risk of poor outcomes should include immunomodulatory drugs such as thiopurine (TP), methotrexate (MTX), and partial enteral nutrition (PEN). Given that MTX is not covered by insurance for CD in Japan, TP and PEN are the primary options. Although TP and PEN are often combined, the efficacy of the TP/PEN combination has not been fully evaluated. To date, long‐term data comparing TP/PEN vs. PEN‐only for maintenance treatment in pediatric CD patients are lacking. This study aimed to determine whether PEN should be combined with TP in pediatric CD patients during initial therapy without biologics.

## METHODS

### Patients

This retrospective study reviewed the clinical records of patients at Osaka Medical and Pharmacological University in Japan from January 2008 to January 2023. Children aged <15 years newly diagnosed with CD who fulfilled the diagnostic criteria of the ESPGHAN guidelines were included.^7^ All children diagnosed with CD had undergone initial diagnostic testing, including upper endoscopy and ileocolonoscopy, capsule endoscopy, or small bowel follow‐through, and histological analysis of multiple mucosal biopsies. The exclusion criteria were as follows: patients with monogenetic disease, inflammatory bowel disease unclassified (IBD‐U), those who did not achieve remission within 12 weeks, those who did not agree to conventional therapy, including those with poor adherence to medication, not taking the prescribed amount of elemental diet (ED) medication recommended by their doctor and those who had not undergone two consecutive endoscopic procedures during the study period. Concomitant therapy was restricted to 5‐aminosalicylates. After the induction of remission, all patients continued with PEN. The effectiveness of the treatment was mainly evaluated via follow‐up endoscopy or capsule endoscopy performed approximately 12–24 months after the start of initial therapy or when symptoms or blood investigation results worsened. In accordance with the STROBE guidelines, all eligible cases were included in the study.^8^ The study protocol was approved by the Ethics Committee of Osaka Medical and Pharmaceutical University (approval no. 2023‐148). Informed consent was obtained via the hospital website, which provided an opt‐out option.

### Treatment

CD management followed international guidelines.^7^ For induction therapy, exclusive enteral nutrition (EEN), steroids, or anti‐TNF therapy is administered according to the severity and patient preferences. Anti‐TNF therapy is recommended for patients with a high risk for poor outcomes, such as those presenting with deep colonic ulcerations on endoscopy, persistent severe disease despite adequate induction, extensive pan‐enteric disease, marked growth retardation (*N* − 2.5 height *Z* scores), severe osteoporosis, stricturing and penetrating disease at onset, and severe perianal disease. For patients without high risk of poor outcomes, EEN or steroids are recommended. All the patients and their legal guardians were offered an informed choice between EEN and steroids. Those who preferred enteral nutrition were initially encouraged to receive it orally; however, if there were concerns regarding tolerance or inadequate intake, a nasogastric tube was used. EEN was administered for at least 6–8 weeks. Steroid therapy was initiated with prednisolone at a dose of 1–2 mg/kg/day and gradually tapered over 3 months. Biochemical remission after induction therapy was defined as a reduction in serum CRP to <0.15 mg/dL. For maintenance therapy, patients who did not receive anti‐TNF therapy opted for TP in conjunction with partial nutritional therapy. Before prescribing TP, patients and their parents were counseled on the possible risks and side effects. The target dose of azathioprine was 1.0–2.5 mg/kg body weight, and that of 6‐mercaptopurine (6‐MP) was 0.5 to 1.5 mg/kg. The dose of TP was modified after initiation, based on dose‐dependent adverse events and laboratory parameters. If no adverse events occurred and the therapeutic effect was insufficient, the dose was increased. ED was initiated immediately after the CD diagnosis. Therefore, we recommend PEN in conjunction with all types of maintenance therapies. PEN involves patients consuming half the amount of their daily allowance of calories through ED and the remaining half from a low‐fat diet (fat: under 20 g/day). In Japan, Elental® (EA Pharma Co., Ltd., Tokyo, Japan) is commonly used as ED. Elental® is available in powdered form, which is reconstituted using tap water to a final concentration of 1.0 kcal/mL (the total energy provided was 300 kcal/80 g of dry weight). Elental® is composed of amino acids, minimal fat, vitamins, trace elements, minerals, and dextrin as the major energy source. As it does not contain selenium or carnitine, these elements were monitored through regular blood sampling, and deficiencies were addressed through dietary guidance. The patients were followed up by a dietician to assess formula tolerance, caloric intake, and weight, between visits as required.

### Data collection

Data were collected retrospectively from the medical records, including information on individual drug adherence, the amount of ED consumed, details of low‐fat food, occurrence of adverse events, blood examination, and anthropometric measurements. All patients had routine outpatient clinic visits at least every 3 months. The disease location was stratified according to the pediatric modification of the Montreal classification.^9^ Clinical disease activity and blood examinations were assessed at baseline and at each visit after initiation of treatment using the Pediatric Crohn's Disease Activity Index (PCDAI). Patients who could not adhere to conventional therapy were considered to have refused conventional therapy and were excluded. To determine the effectiveness of maintenance therapy, endoscopy data, including capsule endoscopy, were collected 12–24 months from the start of the initial therapy, or when symptoms or blood investigation results worsened. Endoscopic evaluations outside this timeframe were not scheduled.

### Outcome measures

The primary outcome was the relapse‐free rates among patients receiving TP/PEN and PEN‐only therapy. Relapse was defined as PCDAI >12.5, and/or Simple Endoscopic Score‐CD (SES‐CD) score >7, and/or Lewis score >135, and/or abnormal CRP levels (CRP >0.15 mg/dL). The secondary outcome was complete mucosal healing (SES‐CD score 0) at 12–24 months.

### Statistical analysis

All statistical analyses were performed using JMP ver. 8 (SAS Institute, Cary, NC, USA). Patient characteristics were described and compared across treatment groups using the chi‐squared test, Fisher's exact test, and Student's *t*‐test. The cumulative relapse‐free rate was estimated using the Kaplan–Meier method and compared using the log‐rank test. Statistical significance was set at *p* < 0.05.

## RESULTS

**FIGURE 1:**
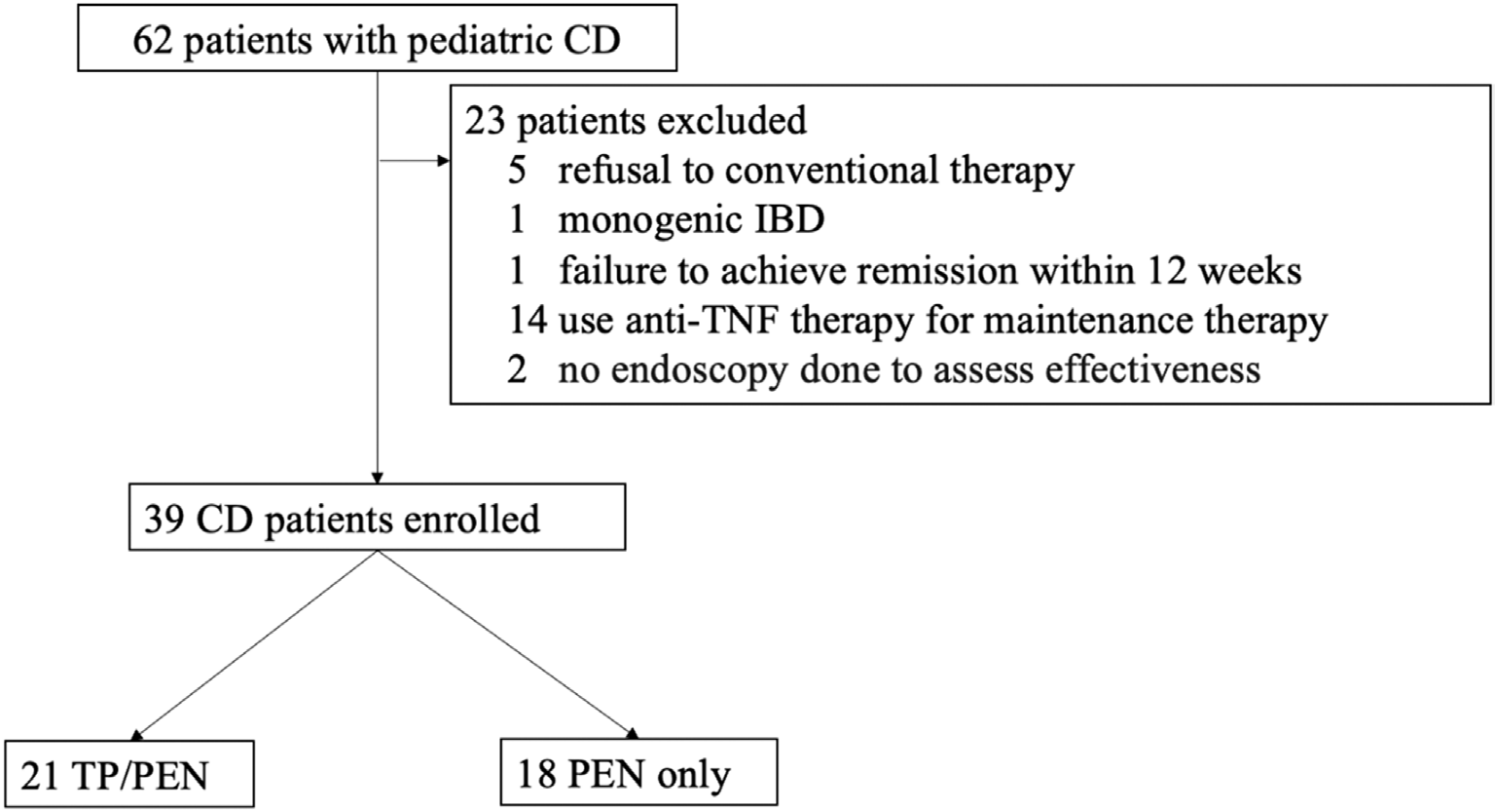
Flowchart of patient selection. Among the patients included in the study, 21 received thiopurine/partial enteral nutrition (TP/PEN), and 18 received PEN‐only. CD, Crohn's disease; IBD, inflammatory bowel disease; PEN, partial enteral nutrition; TP, thiopurine.

**FIGURE 2:**
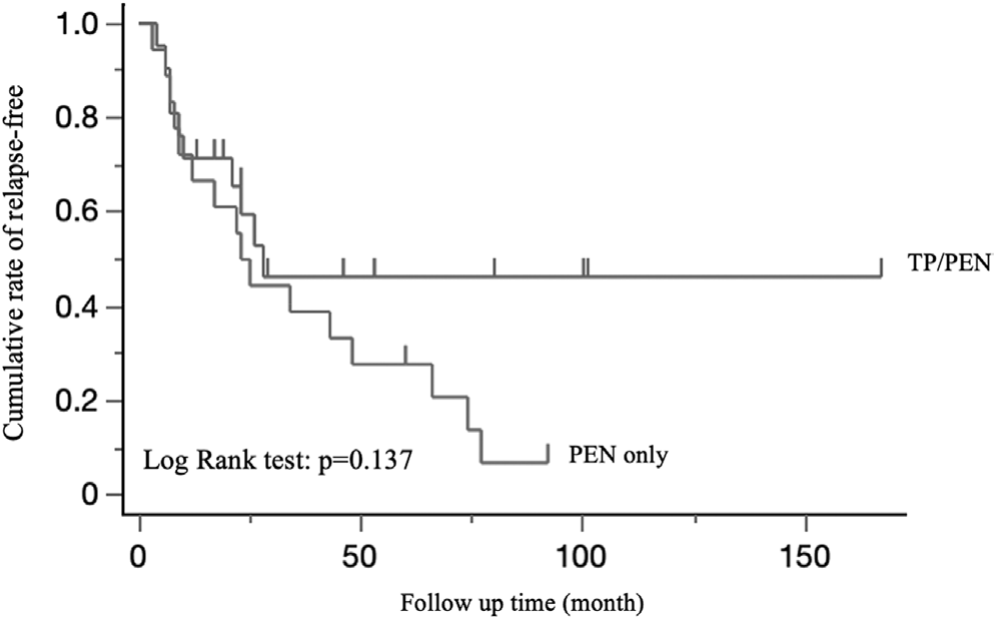
Kaplan–Meier plots showing the cumulative relapse‐free rate between two groups treated with TP/PEN or PEN‐only during the study period. PEN, partial enteral nutrition; TP, thiopurine.

**FIGURE 3:**
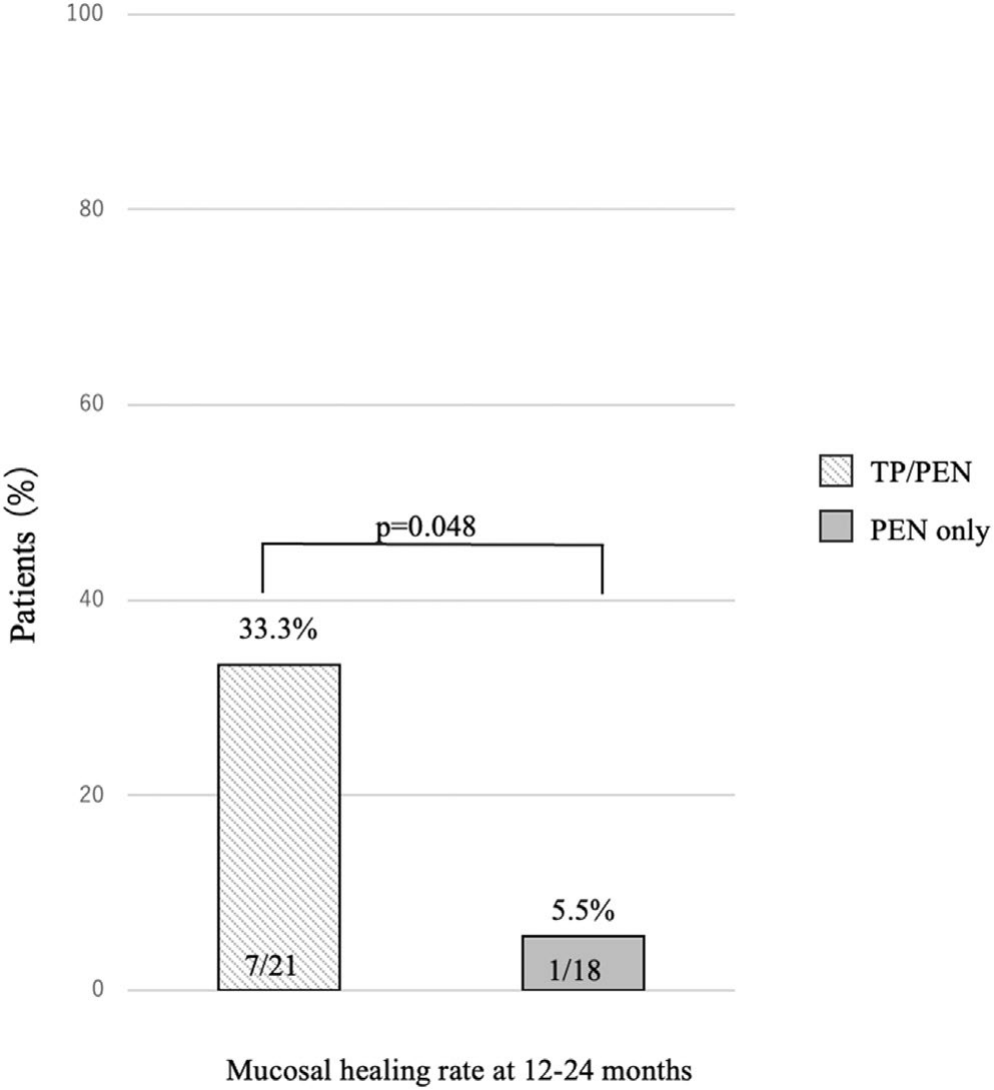
Complete mucosal healing rate at 12–24 months. PEN, partial enteral nutrition; TP, thiopurine.

A total of 62 patients were screened for potential enrollment in the study, of whom 39 met the inclusion criteria and were enrolled. The reasons for exclusion are detailed in Figure 1. Among the enrolled patients, 21 and 18 opted for TP/PEN and PEN‐only for maintenance therapy. The patient demographic characteristics are presented in Table 1. No significant differences in patient characteristics, including age, sex, disease location, or disease activity, were observed between the treatment groups. The amount of ED intake was significantly higher in the PEN‐only group. The median observational period was 61 months (interquartile range [IQR] 37–125). The non‐relapse rates in both groups during the observation period are summarized using the Kaplan–Meier curve (Figure 2). The relapse‐free rates of TP/PEN and PEN‐only at 5 years were 0.46 and 0.27, respectively, which were marginally non‐significant. After 5 years, the relapse‐free rate continued to decline in the PEN group but plateaued and did not decline in the TP/PEN group. Complete mucosal healing at 12–24 months was observed in 7 out of 21 patients (33.3%) in the TP/PEN group and 1 out of 18 patients (5.5%) in the PEN‐only group (*p* < 0.05) (Figure 3). We compared patients who achieved complete mucosal healing with those who did not in the sub‐analysis. We analyzed the following factors: amount of ED, dose of azathioprine (6‐MP was calculated to be 0.6 times that of azathioprine), PCDAI, disease location, albumin levels, and CRP levels.

**TABLE 1:** Characteristics of pediatric patients at diagnosis (*N* = 39).

Characteristic	AZA + PEN (*N* = 21)	PEN (*N* = 18)	*p*‐value
Male sex, *n* (%)	13 (61.9)	12 (66.6)	0.75
Mean age	11.7	10.6	0.59
Mean PCDAI	28.2	32.5	0.62
Mean C‐reactive protein (mg/dL)	4.6	3.9	0.46
Mean Albumin (mg/dL)	3.3	3.2	0.57
*Location*			
Small bowel only	2 (9.5)	5 (27.7)	0.09
Colon only	1 (4.7)	2 (11.1)	
Both	18 (85.7)	11 (61.1)	
Perianal lesions no.(%)	14 (66.6)	10 (55.5)	0.49
Stenosis at diagnosis no. (%)	1 (4.7)	2 (11.1)	0.47
*Induction therapy*			
EEN	9 (42.8)	9 (50.0)	0.88
PSL	11 (52.3)	8 (44.4)	
IFX	1 (4.7)	1 (55.5)	
*Amount of elemental diet intake*			
Mean amount of elemental diet (mL/kg)	30.3	45.0	0.006

Abbreviations: CD, Crohn's disease; EEN, exclusive enteral nutrition; IBD, inflammatory bowel disease; IFX, infliximab; PCDAI, pediatric Crohn's disease activity index; PEN, partial enteral nutrition; PSL, prednisolone; TP, thiopurine.

Significant differences were found only between TP/PEN and PEN.

We also compared the patients who sustained clinical remission with those who did not. We analyzed the same factors and found no significant differences.

Side effects were reported in 3 of 21 patients in the TP/PEN group, including hair loss in one patient and gastrointestinal intolerance in two patients. Hair loss resolved with dose reduction. Two patients reported improvement in nausea after switching to 6‐MP. Almost all patients took ED orally, except one who took ED via a nasogastric tube at night. One patient using PEN had nocturnal enuresis. No other adverse effects were observed in other patients on PEN. In cases of relapse in patients in the TP/PEN group, treatment should be rapidly escalated to biologics without delay with respect to early biologic treatment. For patients on PEN‐only, reinduction therapy was initiated with the addition of TP. If the patient refused to initiate reinduction with steroids or EEN, biologics were initiated.

## DISCUSSION

Nutritional therapy plays a crucial role in pediatric CD and is used in both induction and maintenance therapies. Its advantage is that it causes fewer adverse effects. Given that EEN is as effective as corticosteroids in promoting mucosal improvement,^10^, ^11^, ^12^, ^13^ supporting growth^14^ and bone health, it is recommended as the first‐line therapy to induce remission in pediatric CD. However, few studies have reported on the use of PEN in maintenance therapy. Most reliable studies on PEN have been performed in adults, and these studies show considerable heterogeneity in terms of formulation, dosage, duration of treatment, and disease location. Takagi reported that patients on the half‐ED had a twofold higher rate of clinical remission compared with those on a regular diet during a two‐year follow‐up, suggesting the potential role of PEN in maintaining remission in CD.^15^ Yamamoto et al. performed a systematic review of CD in adults, suggesting the usefulness of PEN for maintaining remission in patients with CD.^12^ Relevant studies on pediatric CD are scarce. A retrospective study by Wilschanski et al.^13^ demonstrated that providing PEN without restriction of the normal diet after successful treatment with EEN was associated with prolonged remission and improved linear growth. In contrast to previous studies suggesting the efficacy of PEN, Knight et al.^16^ reported that PEN was not associated with a significantly decreased relapse rate. Although many studies have shown its efficacy, data available to evaluate the relapse rate and complete mucosal healing rate in pediatric CD are limited.

Although TP, such as azathioprine and 6‐MP, is commonly used in the maintenance therapy of pediatric CD, there is limited data on the optimal timing or indications for its use. The 2014 ECCO pediatric guidelines^17^ recommend it as an option for the maintenance of steroid‐free remission in children at risk of poor disease outcomes. The 2019 Canadian guidelines^18^ suggest TP for maintaining remission in female patients. The 2020 ECCO‐ESPGHAN guidelines^7^ recommend TP for maintaining remission in patients who have achieved remission. In contrast, guidelines for adults recommend against the early introduction of TP therapy to maintain remission in patients with newly diagnosed CD for maintaining remission, restricting its use only for the maintenance of remission in patients with steroid‐dependent CD.^19^ This could be because of the publication of large studies such as the AZTEC study, which did not prove the usefulness of TP.^20^ However, TP is effective for pediatric CD. The ECCO/ESPGHAN guidelines do not clearly indicate when TP should be initiated.

The efficacy of TP is not as high as that of biologics, and its delayed onset of action and potential side effects may explain the limitations for its clinical use. Notably, TP is associated with a higher risk of malignancies, such as lymphoma and non‐melanoma skin cancer, and increased susceptibility to opportunistic infections, which remains a major concern.

The increased risk of lymphoma with TP remains controversial, with large studies yielding conflicting results. Several meta‐analyses have indicated a four‐ to fivefold increased risk of lymphoma in patients with IBD treated with TP than that observed in the general population.^21^ However, it is unclear whether this elevated risk is directly related to medication or disease severity. Another serious complication is hepatosplenic T‐cell lymphoma (HSTCL), a rare form of peripheral non‐Hodgkin lymphoma. HSTCL has a higher predilection in adolescent and young male IBD patients who are treated with TP in combination with anti‐TNF therapy, and it is often fatal.^22^ TP has rarely been implicated in lymphoproliferative disorders and skin cancer in childhood CD, and its absolute risk remains low. Serious infections are rare complications of TP monotherapy. In the SONIC trial, at week 26, only 30% of patients with a median disease duration of 2.4 years achieved corticosteroid‐free remission with azathioprine monotherapy, and only 15% reported mucosal healing.^23^ In the AZTEC trial, azathioprine started within the first 8 weeks after diagnosis of CD was not associated with an increased rate of sustained corticosteroid‐free remission at week 76 compared with placebo. Although many studies highlight the lack of efficacy of TP,^20^, ^24^ many studies demonstrate its effectiveness.^25^, ^26^, ^27^, ^28^, ^29^, ^30^, ^31^ A pediatric study showed that patients in the placebo group experienced significantly higher relapse rates than those in the 6‐MP group, and required more steroids for longer durations.^32^ With over 50 years of global experience, the common and rare adverse effects have been well documented, which is not the case for many recently developed CD drugs.

This study aimed to evaluate the effectiveness of PEN monotherapy or the need for TP in addition to PEN as a maintenance therapy for pediatric CD. To our knowledge, this is the first long‐term study to compare the efficacy of TP/PEN and PEN‐only therapies, including endoscopic evaluation for treatment in pediatric CD patients. Our findings showed no statistically significant differences in relapse‐free rates between the two groups; however, complete mucosal healing was higher with TP/PEN than with PEN‐only therapy. The relapse‐free rates in TP/PEN and PEN‐only groups at 5 years were 0.46 and 0.27, respectively, which were marginally non‐significant. After 5 years, the relapse‐free rate continued to decline in the PEN‐only group, but plateaued and did not decline in the TP/PEN group. This result showed that there was no difference in clinical relapse; however, there may have been a difference if the number of cases was large. Moreover, higher mucosal healing rates indicate that TP/PEN may provide a longer remission in pediatric patients with CD. A major advantage of our study is the comprehensive definition of relapse using clinical scores, normal CRP, and mucosal scores, including the Lewis score for small bowel type, as the definition of relapse. Clinical remission in CD is poorly correlated with endoscopic activity. Relying solely on clinical symptoms may lead to the worsening of mucosal inflammation, and uncontrolled mucosal inflammation contributes to bowel damage and subsequent stricturing or penetrating complications. Given that there is no universally accepted definition of relapse using the PCDAI, we considered PCDAI >12.5, based on the existing literature.^13^ The mucosal endpoint evaluation may be slightly higher, with SES‐CD >7 or a Lewis score > 135 being more realistic.

## LIMITATIONS

Our study had several limitations, including its retrospective nature and small sample size, which limited its statistical power. Due to the nature of observational research, all eligible participants were included without a predetermined sample size. Future studies employing appropriate sample size estimations are necessary to rigorously evaluate the intervention effects. Additionally, we were unable to control for variations in timing with respect to laboratory and endoscopic data. Moreover, NUDT15 polymorphisms are associated with leukopenia, hepatotoxicity, gastrointestinal intolerance, skin rash, and alopecia. Testing before the start of therapy is advocated in international guidelines to avoid myelotoxicity^33^ In Japan, the NUDT15 gene polymorphism test has been covered by insurance since February 1, 2019, but patients who started TP before this date were not tested. TGN levels were not measured because of insurance restrictions. The lack of randomization in this study introduces the possibility of selection bias. For patients with severe CD, anti‐TNF therapy is commonly used for induction and remission. However, for those not receiving anti‐TNF therapy, the decision to use TP for maintenance therapy depends on patient and physician preferences, resulting in treatment bias. From a growth and nutrition perspective, we recommend PEN for all pediatric CD patients, and no patients in this study received TP alone. Finally, fecal calprotectin, a key biomarker in CD, was not assessed. Although fecal calprotectin and CRP are the two most widely used biomarkers of CD, fecal calprotectin generally outperforms CRP.

## CONCLUSION

TP/PEN combination therapy is statistically superior to PEN alone therapy in achieving complete mucosal healing at 12–24 months. In maintenance therapy for pediatric CD, it is difficult to achieve complete mucosal healing with PEN‐only therapy, even in mild cases. Currently, more treatment options are available, and TP/PEN remains a viable option for the treatment of mild‐to‐moderate pediatric CD.

## AUTHOR CONTRIBUTIONS

AY, EK, TN, SN, MO, TO, KI, and TA performed the examination. DN gave statistical advice. EK, AY, and AA designed the study, and EK wrote the manuscript. All authors have read and approved the final version of the manuscript.

## CONFLICT OF INTEREST STATEMENT

Akira Ashida received a lecture fee from Shionogi & Co., Ltd. and received grants from Pfizer Japan Inc., Kyorin Pharmaceutical Co., Ltd., and Pfizer R&D Japan G. K. All other authors declare no conflict of interest.
